# The Contemporary Approach to CALR-Positive Myeloproliferative Neoplasms

**DOI:** 10.3390/ijms22073371

**Published:** 2021-03-25

**Authors:** Tanja Belčič Mikič, Tadej Pajič, Samo Zver, Matjaž Sever

**Affiliations:** 1 Department of Hematology, University Medical Centre Ljubljana, Zaloška 7, 1000 Ljubljana, Slovenia; samo.zver@kclj.si (S.Z.); matjaz.sever@kclj.si (M.S.); 2 Faculty of Medicine, University of Ljubljana, Vrazov trg 2, 1000 Ljubljana, Slovenia; 3 Clinical Institute for Genomic Medicine, University Medical Centre Ljubljana, Šlajmerjeva 4, 1000 Ljubljana, Slovenia; 4 Faculty of Medicine, University of Maribor, Taborska Ulica 8, 2000 Maribor, Slovenia

**Keywords:** calreticulin, chaperone, calcium, myeloproliferative neoplasm, diagnostics, thrombocythemia, artificial intelligence

## Abstract

*CALR* mutations are a revolutionary discovery and represent an important hallmark of myeloproliferative neoplasms (MPN), especially essential thrombocythemia and primary myelofibrosis. To date, several *CALR* mutations were identified, with only frameshift mutations linked to the diseased phenotype. It is of diagnostic and prognostic importance to properly define the type of *CALR* mutation and subclassify it according to its structural similarities to the classical mutations, a 52-bp deletion (type 1 mutation) and a 5-bp insertion (type 2 mutation), using a statistical approximation algorithm (AGADIR). Today, the knowledge on the pathogenesis of *CALR*-positive MPN is expanding and several cellular mechanisms have been recognized that finally cause a clonal hematopoietic expansion. In this review, we discuss the current basis of the cellular effects of *CALR* mutants and the understanding of its implementation in the current diagnostic laboratorial and medical practice. Different methods of *CALR* detection are explained and a diagnostic algorithm is shown that aids in the approach to *CALR*-positive MPN. Finally, contemporary methods joining artificial intelligence in accordance with molecular-genetic biomarkers in the approach to MPN are presented.

## 1. Introduction

In 1951, Damashek was the first to describe four distinct clinical-pathologic entities that later became known as classical myeloproliferative neoplasms (MPNs): chronic myeloid leukemia (CML), polycythemia vera (PV), essential thrombocythemia (ET) and primary myelofibrosis (PMF) [1]. In 1960, Philadelphia (Ph) chromosome was discovered that later led to the identification of *BCR*/*ABL* fusion gene as the main genetic event in the development of chronic myeloid leukemia [2]. It took another 45 years to discover the first mutation in Ph-negative MPNs, the mutation in the Janus kinase 2 gene (*JAK2*), that was first described in 2005 [3,4,5,6,7] and was followed by the discovery of the mutations in the thrombopoietin receptor gene (*MPL*), a year later [8]. In 2013, another gene implicated in the pathogenesis of MPN was revealed [9,10], namely the calreticulin gene (*CALR*), whose role in cancer was recognized previously [11,12,13,14,15]. Recently, *CALR* mutations became an integral part of World Health Organization (WHO) criteria for establishing the diagnosis of Ph-negative MPNs [16]. Today, the detection of these mutations is used in routine patient diagnostic work-up in everyday clinical practice.

The research in the *CALR* gene and its different mutations, is ongoing and several *CALR* mutations and their clinical implications were discovered and thoroughly investigated. The knowledge acquired since 2013 vastly increased and caused a lot of excitement in the scientific community. Here, we present a comprehensive review on the topic.

## 2. *CALR* Mutations

In 2013, Klampfl et al used whole exome sequencing in six patients with PMF who were *JAK2*- and *MPL*-negative and in all of them somatic *CALR* mutations in exon 9 were confirmed, mutations were either deletions or insertions. Secondly, the 896 patients with different types of MPNs were screened for the presence of insertion or deletion *CALR* mutations. *CALR* mutations were observed in patients with ET and PMF [9]. Similar results were obtained by Nangalia et al who analyzed the results of exome sequencing of DNA in 168 patients with MPNs. *CALR* mutations were identified in 26 patients with either ET or myelofibrosis (MF) and non-mutated *JAK2* or *MPL* [10]. There were two most common variants: CALR NP_004334.1:p.L367fs*46, representing a 52-bp deletion (type 1 mutation); and CALR NP_004334.1:p.K385fs*47, which resulted from a 5-bp insertion (type 2 mutation) [9,10]. *CALR* mutations were also recognized in patients with other MPN subtypes and similar diseases, although this is mostly an exceptional event. They were identified in a few patients with chronic myelo-monocytic leukemia and atypical chronic myeloid leukemia [10], myelodysplastic syndrome/myeloproliferative neoplasm (MDS/MPN) [17], unclassified MPN (MPN-U) [18,19] and in rare cases in patients with PV [20]. They were also identified in patients with refractory anemia with ringed sideroblasts and marked thrombocytosis (RARS-t) [21], although this is a rare finding and probably does not occur in patients with strictly WHO-defined RARS-t [22].

Currently, more than 50 *CALR* mutations in exon 9 have been confirmed. Most commonly these are +1 frameshift mutations, either deletions or insertions leading to a change in the C-terminal domain of the calreticulin protein [23]. It seems that only the mutations leading to the +1 frameshift have a pathogenic potential, other mutations are usually germ line variants of *CALR* [23].

Today, mutations that are not type 1 or type 2 are classified according to their resemblance with type 1 or type 2 mutations as type 1-like and type 2-like mutations, respectively [24]. Type 1 *CALR* mutations are more common. In patients with ET type 1 mutation occurs in 55% of patients whereas type 2 mutation occurs in 35% of ET patients. In patients with PMF type 1 mutation is equally more common and occurs in 75% of patients [25]. Classifying mutations as type 1-like and type 2-like mutations carries a prognostic significance and patients with type 1 and type 1-like mutations have a similar predicted survival. Similarly, the prognosis is similar in patients with type 2 and type 2-like mutations. In patients with PMF type 1 and type 1-like mutations have a favorable prognosis compared to type 2 and type 2-like mutations [26].

*CALR* mutations are an important diagnostic marker in patients with suspected MPN which was recognized by the 2016 revision to the WHO classification of myeloid neoplasms and acute leukemia [16] which included *CALR* mutations as one of the major criteria for the diagnosis of ET and PMF. In a retrospective study on 524 patients with suspected MPN our research group confirmed the diagnostic significance of *CALR* mutations in the diagnosis of MPN, however, it seemed that the testing for the presence of *CALR* mutations should only be performed in patients with clear clinical and/or laboratory suspicion for MPN as in other patients *CALR* mutations may be atypical with an unknown clinical significance [27]. At about the same time, a large population-based screening study performed on nearly 20,000 Danish citizens by highly sensitive polymerase chain reaction (PCR) method revealed that type 1 and type 2 *CALR* mutations can indeed be found in patients without confirmed MPN [28]. In fact, in this study, MPN was not confirmed in 30/32 of *CALR*-positive patients. All *CALR* mutations detected were either type 1 or type 2 which are known to cause an MPN phenotype. This study suggests that *CALR*-positive patients are likely to develop MPN even if the disease is not present at the time of *CALR* mutation detection. Type 1 and type 2 mutations may therefore represent a pre-MPN state with a potential to develop into overt MPN over time [28].

## 3. The Calreticulin Protein

**Figure 1:**
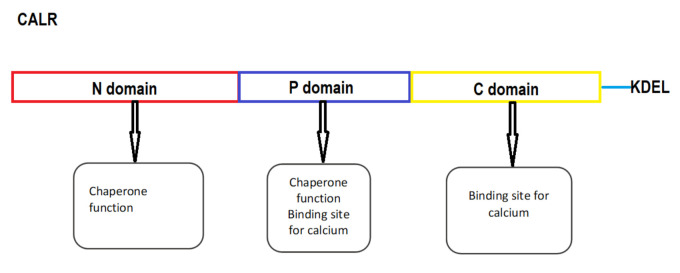
The schematic structure of CALR. KDEL, endoplasmic reticulum-retention signal.

Calreticulin (CALR) was first recognized by Ostwald and MacLennan in 1974 [29]. It is a 46 kDa protein with a role in many cell processes in the endoplasmic reticulum (ER) as well as in the cytoplasm. Two major functions of CALR are intracellular calcium homeostasis and chaperone function. In the ER it binds calcium and thereby affects its intracellular homeostasis. As a chaperone it enables the proper folding of proteins [30]. CALR has three domains: a globular N-domain, an extended proline rich P-domain and an acidic C-domain. Each domain has a specific function. Both the N- and the P-terminal domains are responsible for the chaperone function of CALR, the N-domain contains the binding sites for polypeptides and carbohydrates and the P-domain contains secondary binding sites. C-domain consists of a large number of negatively charged residues that are responsible for the calcium regulating function of the protein. The C-terminal domain also contains an ER retention signal (KDEL) which prevents the protein from leaving the ER [31]. CALR binds more than half of the ER luminal calcium [32] which is bound by the C-terminal domain with low affinity and high capacity. It also contains a high affinity and low-capacity binding site for calcium in the P-domain [31]. Calcium has a significant effect on the structure and conformational stability of CALR, making it more compact and stable [33]. Recent studies show that the two major functions of CALR are tightly connected as the binding of calcium to CALR may have an impact on its chaperone activity as well as calcium storage [34]. CALR seems to be a structural “chameleon” protein with multiple different structures involved in distinct functions [35]. It has a role in the immune response as it enables the assembly and cell surface expression of major histocompatibility complex (MHC) class I molecules and thereby cytotoxic T cell recognition of antigens. Recently, is was shown that the CALR C-terminal domain has a role in the embryonic development of ventricular myocardium [36]. On the surface of living cancer or dying cells it initiates anti-tumor (or antioncogenic) responses by promoting phagocytosis [37]. On the other hand, the most evident oncogenic properties of CALR are characteristic somatic mutations leading to a change in the C-terminal domain and the occurrence of MPN [38]. The structure of CALR is presented in Figure 1.

## 4. Mutant CALR

*CALR* mutations are gain-of-function mutations leading to cytokine independent cell growth [9,10]. CALR mutants obtain a novel C-terminal domain rich in positive amino acids and lacking the ER-retention KDEL sequence [39]. The oncogenic properties of CALR mutants are, in fact, related to this novel C-terminal domain and are not a consequence of a specific sequence of the C-terminal domain but are rather linked to its positive electrostatic charge [40,41] with MPN transformation manifested through the physical interaction between the positive electrostatic charge of the mutant C-terminal domain and the thrombopoietin receptor (TpoR/MPL) [40]. It seems that the threshold of positive charge in the mutant C-terminal domain influences the binding of mutant CALR to MPL as well as the activation of MPL signaling [42]. Mutant CALR binds to the extracellular domain of MPL whereas its intracellular domain is required to activate signaling [42]. This binding is followed by the constitutive ligand independent activation of Janus kinase 2 and signal transducer and activator of transcription 5 (JAK2/STAT5) signal pathway [41,43,44] which results in dysregulated megakaryopoiesis and the occurrence of thrombocytosis [41,45]. CALR mutants have an autocrine function and recognize only the immature form of MPL [18,23]. It is mandatory for the activation of MPL that the CALR mutant lacking the ER-retention KDEL sequence enters the ER secretory pathway. Namely, CALR mutants in lack of the signal peptide are unable to activate STAT5 transcriptional activity [46]. It was shown that CALR mutants interact with each other through mutant specific sequences to form homo-multimeric complexes which is required for MPL binding and activation [47]. The activation of MPL occurs only after the ER compartment. CALR/MPL complexes are present in the Golgi apparatus and are then transported to the cell surface together. The interaction between MPL and CALR is based on the link between N-sugars of the MPL and the lectin binding domain of CALR [46]. CALR mutants can enable the traffic of not only mature MPL but even defective MPL to the cell surface and, as such, act as rogue chaperones [46]. It is necessary for MPL to be located on the cell surface to enable the mutant CALR-dependent activation [48] and CALR mutants can be detected in the plasma of CALR-positive patients [36]. In mouse models CALR release was confirmed with extracellular CALR performing immunomodulatory properties and inhibiting the phagocytosis of dying cancer cells [49].

Additionally, CALR mutants exhibit their oncogenic role through altered epigenetic regulation. As a modulator of the regulation of gene transcription CALR was recognized more than twenty years ago [50,51] with its nuclear localization in all the cell types confirmed later [52] and more recently even in the megakaryocytes [53]. CALR can in fact, act as a chaperone from the cytoplasm to the nucleus. By binding to the transcriptional factor FLI1 and altering its cellular localization CALR mutants affect transcriptional regulation and stimulate the expression of MPL [54]. This is an important oncogenic mechanism as the clonal advantage by mutant CALR is likely promoted only in those hematopoietic progenitor cells that express MPL [55]. CALR mutants can even promote the ability of CALR itself to bind to the MPL promoter. The end result is an increased MPL/JAK2 activation by enhancing the expression of MPL in CALR mutant cells [54].

Another contributing factor to the occurrence of MPN phenotype is the alteration of calcium storage by CALR mutants. Defective interactions between mutant CALR and other proteins (ER protein 57, stromal interaction molecule 1) result in spontaneous outflow of calcium in the cytosol. This leads to an increased activation of JAK2/STAT5 pathway and the proliferation of megakaryocytes [56]. It seems that CALR mutants exhibit their oncogenic potential by affecting both principal roles of CALR, the chaperone function as well as calcium homeostasis.

**Figure 2:**
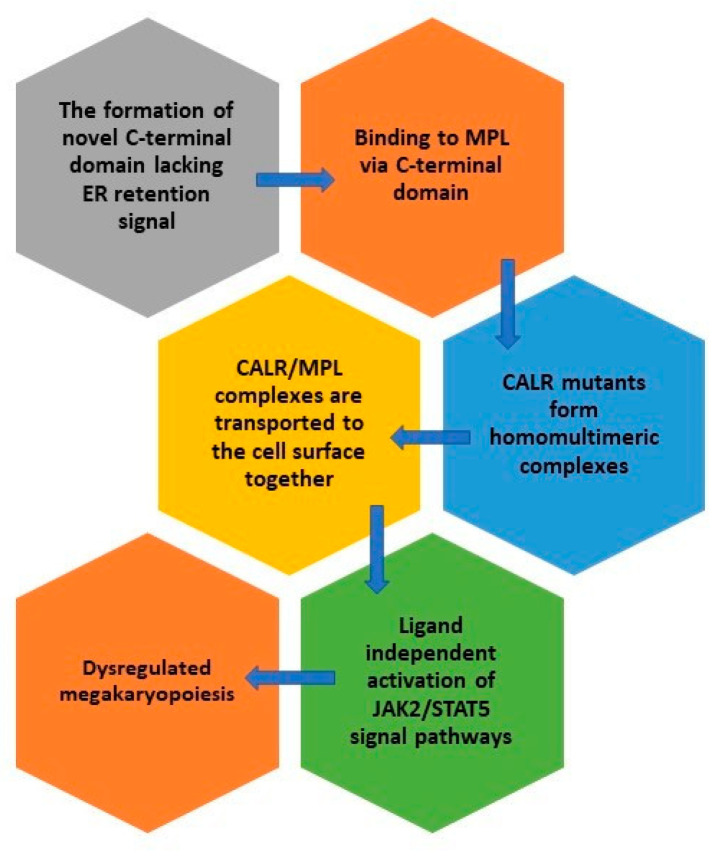
The pathogenic effects of CALR mutants.

Moreover, common CALR mutants induce different effects on hematopoiesis. Overall, del52 activity is more potent than ins5 in promoting hematopoiesis and all features are amplified by homozygosity [57,58]. Studies on animal models have shown that del52 mutants develop a more severe thrombocytosis than ins5 CALR mutants [57]. Other phenotypic changes greater in del52 mutants include leukocytosis, splenomegaly, bone marrow hypo-cellularity and the amplification of the megakaryocytic lineage. Thrombocytosis appears due to both, an increase in the size and the number of megakaryocytes. Other factors influencing the magnitude of thrombocytosis are the amount of CALR mutants and the ratio of CALR mutants to CALR wild type (wt) [57]. The pathogenic effects of CALR mutants are described in Figure 2.

The deeper understanding of the exact mechanism underlying the development of MPN phenotype in *CALR*-positive patients led to consideration of novel therapeutic strategies. Examples are inhibitors of CALR-MPL binding [54] and vaccines with mutant CALR epitopes [59].

## 5. Detection of *CALR* Mutations and In-Depth Mutational Analysis

Alongside traditional Sanger sequencing which has relatively poor sensitivity (limit of detection (LoD) of 10 to 20%) [60], several other molecular genetic screening techniques have been published that are used for the detection of *CALR* mutations in MPN patients (Table 1). Among them, PCR followed by fragment length analysis [60,61,62,63,64] and high-resolution melt (HRM) [60,65,66,67,68] methods are widely used due to their simplicity, low cost, rapidity and the detection of almost all the relevant *CALR* frameshift mutations with a relatively high sensitivity (LoD of 1 to 5%) (Table 1). These techniques have sufficient sensitivity to detect high levels of *CALR* allele burden in most MPN patients (greater than 10%), especially if the test is performed on a deoxyribonucleic acid (DNA) sample of peripheral blood or bone marrow granulocytes [69]. However, both approaches need to be followed by Sanger sequencing for correct genotyping of the *CALR* mutations [27,70]. Recently, quantitative real-time and digital PCR with the LoD of below 1% (Table 1) were introduced [28] for detecting the most common *CALR* variants (mutation types 1 and 2) as a sensitive screening diagnostic method [71] or measuring minimal measurable disease (minimal residual disease or MRD) after allogeneic hematopoietic stem cell transplantation or other type of treatment in patients with MPN [62,71,72]. Although these two methods enable a rapid and extremely sensitive detection of type 1 and type 2 *CALR* mutations, their use in everyday clinical practice is limited. The major limiting factors are an unclear association between *CALR* allele burden at diagnosis and the MPN disease phenotype, as well as an unclear clinical value and prognostic significance of MRD status during or after currently available treatment options [69].

Depending on the type of mutation, the mutant CALR retains a varying amount of the negatively charged amino acids from non-mutant calreticulin [73]. Type 1 *CALR* mutation eliminates almost all the negatively charged amino acids whereas type 2 retains approximately half of them. The other (non-type 1 or 2) frameshift *CALR* mutations have been classified as either type 1-like, type 2-like, or indeterminate, based on their structural similarities to the classical mutations and using a statistical approximation algorithm (AGADIR) of preservation of the secondary protein structure α helix close to the wild type CALR with the clinically established cut-offs for type 1/type 1-like mutations (an AGADIR scale of 26% or less) and type 2/type 2-like mutations (an AGADIR scale of 30% or more). AGADIR is an online tool available at agadir.crg.es, (last accessed March 13, 2021) where 31 unique amino acid sequences that are altered by the *CALR* specific mutation can be entered to determine the helix propensity score (an AGADIR score). In rare circumstances when the AGADIR score of the *CALR* mutation of interest is out of the scale for type 1/type 1-like or type 2/type 2-like, the term “indeterminate” is proposed. In these patients *CALR* mutation type cannot be used as a prognostic marker and reliance on other prognostic markers should be used [73].

Moreover, whole genome sequencing, which allows sequencing of the entire human genome, whole exome sequencing, which covers the coding regions (exons) of the approximately 3.0% of the total human genome (human reference genome GRCh38) [74] and targeted next-generation sequencing (NGS), which allows the sequencing of a certain number of genes (NGS panels) have been applied for the comprehensive genomic or genetic profiling of patients with MPN. These methods reveal new insights into the genomic basis of MPN including the discovery of new driver and non-driver mutations, thereby improving the diagnosis, prognosis, treatment, or prediction of treatment in patients with MPN [69,75,76,77,78]. NGS (short-read NGS sequencing) is a high-throughput method concomitantly detecting different types of genetic variations such as substitution of nucleotides, small deletions/insertions (indels), structural changes and copy number variations in many patients in the same run [78,79]. In the recent years, medical laboratories all around the globe implemented this method into a routine everyday use due to a lower cost of NGS test per sample, shorter workflow times from sample to result, improved bioinformatics tools for analysis and interpretation of NGS results and available recommendations for NGS method validation and result interpretation [79]. It was confirmed that the NGS panels are sensitive in detecting *CALR* mutations with a limit of detection (LoD) of 1% to 5% (Table 1) [75,79]. Its sensitivity depends on the size of the gene target panels, methods for target enrichment, sequencing depth and bioinformatics tools that allow the precise characterization of *CALR* indels in MPN patients [80]. The latter would be of particular importance because technical capacity for detecting variant types differ between commercial or custom made NGS panels [78]. Therefore, careful design of the validation protocol is necessary to ensure that all relevant parameters are addressed as efficiently as possible before implementing NGS assay into routine practice [79]. Nonetheless, it could happen that the precise variant characterization including the larger frameshift *CALR* variants cannot be defined by NGS test [79,81]. In this case, the visual exploration of genomic data of the aligned reads by The Integrative Genomics Viewer (IGV) and other, orthogonal molecular genetic test can greatly reduce the risk of false positive results [79,81].

By the careful selection and implementation of clinically important genes associated with MPN into the NGS panels, this method can assist even in the diagnostic process of patients with suspected MPN and lack of any of the three main driver mutations. NGS can detect the most frequent additional mutations which aids in determining the diagnosis of MPN [82,83]. In the future, the diagnostic approach to patients with suspected MPNs could change, moving from the current cascade testing approach, based on mutational frequencies in *JAK2*, *CALR* and *MPL* and clinicopathological variables of specific MPNs, to the more comprehensive and informative approach, relying on the NGS testing [60,69,75,78,83]. Nonetheless, according to our knowledge this method is not yet equally available worldwide.

Finally, molecular diagnostic testing is a valuable and reliable tool in assessing patients with MPN, however, it cannot entirely replace bone marrow biopsy. Histologic evaluation of bone marrow is still required to distinguish between different MPN subtypes and to diagnose patients who are negative for the most frequent driver mutations or additional mutations in the genes associated with MPN [84].

## 6. The Clinical Value of *CALR* Mutations

In the current clinical practice, the vast majority of *CALR*-positive clinical conditions are ET and PMF. The diagnostic approach to patients with ET and PMF is based on the 2016 WHO criteria [16]. From a clinical point of view most ET patients are referred to a hematologist due to accidentally identified elevated platelets in their blood counts. They can present with erythromelalgia, rarely with enlarged spleen and commonly with thrombotic events [89]. The risk for thrombotic complications in patients with ET exceeds 20% [90]. In PMF, patients often present with hepatosplenomegaly, abdominal tenderness in the left upper quadrant, early satiety, fatigue and bone pain [91]. Blood counts in patients with PMF show anemia and variably peripheral blood leukoerythroblastosis [92]. An important hallmark of PMF is extramedullary hematopoiesis which leads to organomegaly and has its origins in the release of bone marrow precursor cells into the circulation as well as overproduction of cytokines that stimulate hematopoiesis and represent a potential therapeutic target [93,94]. Moreover, thrombotic events can be a presenting feature and occur nearly as often as in patients with ET [95].

A bone marrow biopsy is vital for distinguishing between different MPN subtypes and should be performed in all patients. The histologic focus is mainly on megakaryocyte morphology and bone marrow fibrosis involvement. This can help in distinguishing between ET, pre-fibrotic MF and overt MF which all have different prognosis and treatment approach [96]. In all patients with suspected ET or PMF molecular diagnostic tests revealing driver and non-driver mutations need to be performed that can aid in the diagnosis and management of the disease.

Up to this date, driver mutations in the three previously mentioned genes were recognized in ET and PMF patients. In the remaining 10 to 15% of patients with ET or PMF none of these driver mutations are recognized; these patients are referred to as triple negative [97]. The role of driver mutations was elucidated in the recent years. The lower *JAK2* allele burden was shown to be associated with poorer survival in PMF [98,99]. *CALR*-positive patients compared to *JAK2*-positive patients are younger, have higher platelet counts and are less likely to be anemic, thrombocytopenic, require transfusions or display leukocytosis [100]. They also have a lower risk of progressing to myelofibrosis or acute leukemia and developing thrombotic complications [101,102]. Their leukemia-free and overall survival is superior [100,101]; however, the survival advantage is restricted to type 1/type1-like *CALR* mutations [103,104]. In patients with ET *CALR* mutations are associated with younger age, lower hemoglobin level, white blood count, platelet count and erythropoietin level compared to *JAK2*-positive ET patients. *CALR*-positive patients with ET have no polycythemic transformation, a similar risk of myelofibrotic transformation and a significantly lower risk of thrombosis compared to *JAK2*- positive patients [101]. The incidence of splenomegaly between *JAK2*- and *CALR*-positive ET patients is similar [105]. These findings represent a foundation for a risk-based therapeutic approach to patients with ET with *CALR* mutations representing favorable mutations with a lower risk of thrombotic complications and less need of antithrombotic therapy especially in younger patients with no thrombotic history [106].

Additionally, non-driver mutations affect the disease characteristics. With the aid of NGS several novel molecular biomarkers were identified, some of them carrying a prognostic significance [82]. Some examples are mutations in the genes *LNK* (*SH2B3*), *TET2*, *DNMT3A*, *IDH1/2*, *CBL* and *ASXL1* and atypical *JAK2* and *MPL* mutations [107,108,109]. Other genes important in the pathogenesis of MPN are DNA methylation genes (*TET2*, *DNMT3A* and *IDH1*) and chromatin structure regulation genes (*EZH2*, *ASXL1*) [110]. In a study by Agarwal et al 12% of *JAK2*-positive patients had additional mutations in the genes *TET2*, *ASXL1* and *SF3B1*. Additional mutations were also present in up to 10% of *CALR*-positive patients, in the genes *TET2* or *ASXL1* [111]. Revolutionary, Grinfeld et al developed a prognostic model based on the sequencing of 69 myeloid genes. This model considered 63 clinical and genetic variables and created a personally tailored clinical prognosis representing a personalized approach to patient prognosis in MPN [77]. Today, routinely, apart from the main three driver mutations (*JAK2*, *CALR*, *MPL*), other mutations in the common myeloid genes can be determined by using the NGS technology which can confirm the accuracy of the diagnosis and provide prognosis [80]. Currently, mutational information on the presence of non-driver mutations is incorporated into a new prognostic model for ET, the Mutation-Enhanced International Prognostic Scoring System (MIPSS). Mutations in the following genes: *SRSF2*, *SF3B1*, *U2AF1* and *TP53* are considered unfavorable [112]. This model is based on a large cohort of ET patients with unfavorable mutations showing a survival disadvantage [113]. The implementation of this model into clinical practice is yet to be elucidated. The prognostication of PMF, on the other hand, went through several stages as the knowledge of the condition increased. The current most often used prognostic system is a dynamic prognostic model (DIPSS) based on age, the hemoglobin level, leukocyte and blast count and the presence of constitutional symptoms [114]. It can be used at any stage of the disease. The implementation of driver and non-driver mutations into prognostic systems in addition to the karyotype, sex-adjusted hemoglobin levels and focus on transplant population led to the development of the most advanced prognostic systems: mutation-enhanced international prognostic scoring system for transplant-age patients (MIPSS70), the karyotype enhanced MIPSS70 (MIPSS70+ version 2.0 (MIPSSv2)) and the genetically-inspired prognostic scoring system (GIPSS) which are relevant in daily patient management [115,116,117].

**Figure 3:**
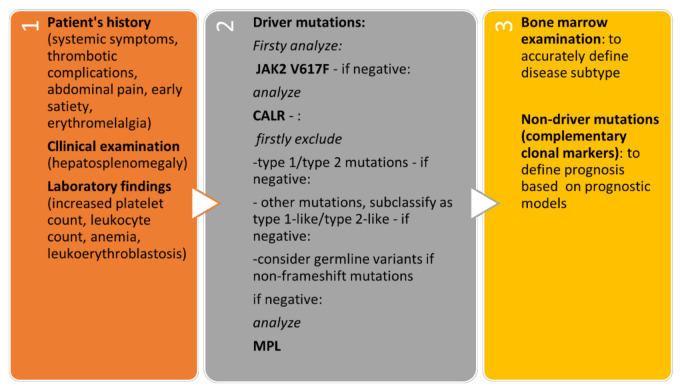
Algorithmic approach to patients with suspected *CALR*-positive MPN.

The algorithmic approach to patients with suspected *CALR*-positive MPN is presented in Figure 3**.**

## 7. Machine Based Learning and its Role in MPN

Artificial intelligence (AI) methods are being implemented widely in all fields of medicine such as diagnostics, disease prognosis and treatment decision, including in the field of MPN [118]. Machine learning is an application of AI that provides the capability to automatically learn and improve from experience without being explicitly programmed [119]. The process is an extension of statistical methods leading to predictions and automatic identification of patterns ending in performing tasks beyond human capabilities [120].

We performed a systematic review of English articles in PubMed. The search terms included “artificial intelligence”, “expert system” and “machine learning” in combination with either ”essential thrombocytosis”, “polycythemia vera” or “myelofibrosis”. We identified 9, 8 and 13 articles for ET, PV and MF, respectively. After reviewing the results only two adequate papers per each condition remained for further analysis with one article referring to all three MPNs.

In ET, PV and MF Sirinukunwattana et al. looked into morphological assessment of bone marrow biopsies by using an approach for the automated identification, quantitative analysis and abstract representation of megakaryocyte features using reactive/nonneoplastic bone marrow samples of patients. Their analysis enabled the diagnosis of MPN with a high predictive accuracy and showed potential to discriminate between important MPN subtypes. The AI approach could significantly affect and complement routine diagnostics or the assessment of disease progression/response to treatment [121].

Guo et al. used bioinformatics analysis to identify differentially expressed genes (DEGs) in ET associated thrombosis. They used Cytoscape software with cytoHubba and MCODE plugins. With their analysis, they identified DEGs and hub genes that interacted with CD34+ cells and neutrophils that may predict an increased risk of thrombosis in patients with ET [122].

Liu et al. used PV to performed various molecular complexes detection using the Clustervize plugin while gene ontology-enrichment analysis of the biological pathways, molecular functions and cellular components of the selected molecular complexes was performed using the BiNGo plugin. In their work, they identified five molecular complexes associated with the JAK-STAT signal transduction pathway, neurotrophic factor signaling pathway and Wnt signaling pathway, which were correlated with chronic myeloid leukemia and acute myeloid leukemia [123].

Using MF as model Xu et al. obtained MF gene expression profiles, known pathogenic genes and protein-protein interactions to identify differentially expressed genes (DEGs) and seed genes. A new network was constructed using the seed genes and their adjacent DEGs within the protein-protein interaction network. The study predicted 10 candidate pathogenic genes and several signaling pathways that may be related to the pathogenesis of PMF [124].

Artificial intelligence in MPN is at an early stage of development. We identified a small number of publications, mainly in the diagnostic field and modelling of cellular mechanisms. However, with the AI developments in other fields of hematology it is very likely we will see its further implementation into disease prognostication, treatment strategy selection, warning of complications and more.

## 8. Conclusions

*CALR* mutations have a rather short history in hematology; however, due to the rapid development in the knowledge of their role in MPN, it seems their impact is large. The understanding of *CALR* mutation types led to the development of different assays aiding in their detection. Currently, all the pathological mechanisms causing a diseased phenotype in *CALR*-positive MPN were not elucidated yet. Nonetheless, it is evident that mutated CALR has many different cellular pathogenic pathways. By binding to MPL and promoting the survival of even misfolded MPL it enables its activation and consequently a constitutive ligand independent activation of JAK2/STAT5 intracellular signaling leading to dysregulated megakaryopoiesis. CALR mutants exhibit their oncogenic potential additionally through homo-multimerization, altered epigenetic regulation and defective calcium storage. As their role in the human cell becomes clearer, novel therapeutic strategies are evolving with potential impact on patients’ outcome. Lastly, in the future the knowledge of the molecular-genetic basis of the disease and their clinical value could become part of the machine learning systems designed to properly diagnose and predict outcome in patients with MPN.

## Figures and Tables

**Table 1:** Comparison of different molecular genetic tests for *CALR* mutations detection in patients with suspected myeloproliferative neoplasm.

Method	Advantage	Critical Remarks	Sensitivity	Reference
Sanger sequencing	Known and unknown genetic variant detection.	Low sensitivity; Not quantitative; Moderate cost.	10 to 20%	[9,10,60]
PCR and fragment analysis	Known and unknown genetic variant detection; Qualitative and quantitative; Simple to perform; Low cost and rapid.	Moderate to low sensitivity.; Preferential amplification of shorter amplicons may lead to over- or underestimation of the mutant allele burden; Sanger sequencing needed for correctly genotype the *CALR* variants.	1 to 10%	[9,60,61,72,80,85]
High-resolution Melt	Known and unknown genetic variant detection; Simple to perform; Low cost and rapid.	Moderate to low sensitivity; Not quantitative; Sanger sequencing needed for correctly genotype the *CALR* variants.	1 to 5%	[60,66,67,68]
Quantitative PCR (real-time PCR) (qPCR)	High sensitivity; Quantitative; Rapid.	Detects only target genetic variants; Moderate cost.	0.01 to 1%	[60,62,71,86]
Digital PCR	High sensitivity; Quantitative; Rapid.	Detects only target genetic variants; Moderate cost.	0.01 to 1%	[28,72,86,87,88]
NGS	Known and unknown genetic variant detection; Simultaneous screening of multiple genes in multiple samples.	Complex genetic variants and large indels need in some instances confirmation by alternate molecular genetic methods; Complex workflow and result interpretation; Moderate to high cost.	1 to 5%	[60,75,80]

Abbreviations: PCR, polymerase chain reaction; NGS, next generation sequencing.

## Data Availability

Did not report any data.
